# A metric of knowledge as information compression reflects reproducibility predictions for biomedical experiments

**DOI:** 10.1098/rsos.241446

**Published:** 2025-07-09

**Authors:** Daniele Fanelli, Pedro Batista Tan, Olavo B. Amaral, Kleber Neves

**Affiliations:** ^1^ Theoretical and Empirical METaknowledge (TEMET) lab, School of Social Sciences, Heriot-Watt University, Edinburgh, UK; ^2^ Department of Methodology, London School of Economics and Political Science, London, UK; ^3^ Vrije Universiteit Amsterdam, Amsterdam, The Netherlands; ^4^ Universiteit van Amsterdam, Amsterdam, The Netherlands; ^5^ Federal University of Rio de Janeiro, Rio de Janeiro, Brazil

**Keywords:** metascience, metaresearch, reproducibility, complexity, information compression, philosophy of science

## Abstract

Forecasting the reproducibility of research findings is one of the key challenges of metascience. Above-chance predictions have mainly been achieved by pooling the subjective ratings of experts, and how these predictions are formed remains to be understood. Here, we show that reproducibility forecasts made for the Brazilian Reproducibility Initiative (BRI), a large-scale replication of experiments in the life sciences, are significantly correlated with *K*, a principled metric of knowledge as information compression. For each study in the BRI sample, we calculated *K* by dividing the effect size, measured in bits of Shannon entropy, by the descriptive length (a proxy of the complexity) of the study’s methodology, calculated as the optimal Shannon encoding of a conceptual graph representing the replication protocol. We found that experts’ predictions about reproducibility were statistically associated with *K* values and with the complexity of protocols. This relation was robust to controlling for study methodology and other possible confounding factors. These results suggest that expert raters partially judge the reproducibility of findings by assessing the ratio between the information yielded and the information required by a study, and they support the hypothesis that scientific knowledge may be understood and studied through the lenses of information compression.

## 1. Introduction

Several metaresearch studies have sought to estimate the expected reproducibility of published results, by sampling articles in the literature and having different labs attempt to replicate the original findings on new data. The interpretation of these studies is surrounded by uncertainty, due to methodological differences and lack of consensus on how to measure reproducibility [1]. Nevertheless, it is generally agreed that published findings have variable reproducibility: on average, the effect size measured across replications is smaller than in the original report and a non-negligible proportion of replications fail to reject the null hypothesis despite high statistical power [2–6].

Predicting how well individual studies will replicate is therefore a key metascientific objective, and it has proven to be challenging [7,8]. A few study characteristics, including the type of method and the strength of the original evidence, appear to be associated with replication success, at least in the social sciences [9], but they explain a modest amount of variance and may not generalize across research fields. The expertise of replicators appears to correlate with replication success, but mainly because expert replicators choose studies that are more likely to replicate [10]. Deep learning methods were able to predict the presence or absence of a statistically significant effect in the replication with 68% accuracy based only on the original article’s text, and 71% when statistical information about the original effect size was included [11]. However, the theoretical significance of features used by deep learning is unclear and, due to the limited number of studies available as training and testing samples, these results may have limited generalizability.

The highest predictive accuracy to date has been obtained by pooling the subjective predictions of human raters. Several systematic replication studies have collected such predictions, usually by using both surveys and prediction markets. According to a recent review, survey scores predicted replications with an average 66% accuracy and had an average correlation of r=0.564 with replication effect size, while prediction markets are slightly more successful (on average 73% and r=0.581, respectively) [8]. While relatively high, the performance of expert forecasts is far from perfect and likely to vary depending on the expertise and understanding of participants [12]. Moreover, most of these prediction studies have been concentrated in the social sciences, and their generalizability to other areas is unclear.

How experts formulate their predictions is not known. Prediction markets yield no explicit data about what information is used and how it is processed to shape the final predictions. Surveys may include open questions asking experts to explain what led to their decisions, but much of the processing that underlies such decisions may be unconscious or based on ‘tacit’ expertise that may not be articulated by participants.

We hypothesized that a theoretical framework and a metric that were proposed a few years ago to measure knowledge [13] might help explain how reproducibility forecasts are formed, and tested this hypothesis on data collected by the Brazilian Reproducibility Initiative (BRI). The BRI started in early 2018 as an effort to evaluate the reproducibility of Brazilian biomedical science, following the blueprint of previous multi-centre efforts. Studies to be replicated were chosen as a representative sample of three methods commonly used in Brazilian biomedical science [14]. In addition to assessing directly the reproducibility of these studies, the BRI collected expert predictions about their reproducibility. These predictions are the empirical data on which we tested our hypothesis.

### 1.1. Information compression as a framework for metascience

What we shall henceforth refer to as ‘Information Compression Theory’ (ICT) is a principled approach to measuring, studying and predicting knowledge and other cognitive phenomena, proposed as a candidate paradigm for metascience [13]. At the core of ICT is the notion that knowledge is essentially a manifestation of information compression. This is a philosophical position that has a very long history—for example, it was proposed *ante litteram* by the physicist Ernst Mach (1838−1916) [15]. With the development of classic and algorithmic information theory [16,17], it has found theoretical and methodological applications, particularly in statistics (e.g. Akaike’s Information Criterion [18], Rissanen’s Minimum Description Length principle [19]) and computer science (e.g. Kolmogorov complexity [20]). In the last few decades, information compression has been revived as a candidate foundational principle of scientific inference and cognition (e.g. [21–23], see further discussion in [13]).

ICT proposes to apply these ideas to metascientific problems, extending its philosophical, theoretical and methodological implications. Philosophically, it proposes to conceive of any scientific claim (indeed, any form of knowledge) as the property of a system, in which both the phenomena studied and the theories and methods used to study them are describable in terms of structures of concepts and relations [13,24]. Theoretically, ICT proposes that important properties of knowledge claims are captured by a metric, K, that relates the information yielded by the claim to the information needed to describe the entire system. The mathematical structure of K is one of the key distinctive features of ICT, as it includes multiple information quantities and particular mathematical relations between them that, to the best of our knowledge, have not been previously proposed in the literature in this form (see [13] for a discussion). Methodologically, ICT proposes to use a novel combination of knowledge representation (e.g. [25,26]) and minimum description length techniques (e.g. [27,28]) to quantify the denominator of K (see [24]).

Adopting a compact notation, K may be succinctly expressed as:


(1.1) $$\begin{array}{lcr} & K=\frac{I}{D}\text{, } & \end{array}$$

in which:

— I is the amount of information yielded by the study. In particular, $I=n_{y}\left[H(Y)-H(Y|X,\tau )\right]$ is Shannon’s mutual information [17] between the random variable constituting the *explanandum* Y (i.e. the uncertainty about the state of the world that the knowledge claims to reduce) and the *explanans* X (i.e. the set of measurements, parameters and conditions that need to be obtained in order to get the answer), conditioned on a structure τ (i.e. the structure that defines the relations between Y and X), and multiplied by the number of times $n_{Y}$ that the knowledge can be applied (e.g. the total number of objects or events to which the reduction in uncertainty may be applied). Note that the random variables Y,X may represent any joint distribution of random variables, in which case their Shannon entropy H(Y),H(X) is merely the sum of the conditional entropies of the composing random variables (i.e. $H(Y_{1},Y_{2},...Y_{m})=\sum H(Y_{i}|Y_{i-1}...Y_{1})$). Therefore, Y,X may represent any number of variables with any level of correlation between them.
— D is the amount of information needed to describe the system, i.e. the system’s (descriptive) complexity. In particular, $D=n_{y}H(Y)+n_{x}H(X)+\log\;1/p(\tau )$, in which $n_{y}H(Y)$ expresses the total amount of information in the explanandum, $n_{x}H(X)$ the total information that needs to be collected (i.e. parameters that are measured or fixed) and log⁡1/p(τ) is the description length of the structure, calculated as the Shannon encoding of the graph (more accurately, it is a nested, labelled hypergraph) that describes the structure (see Methods and [24] for further details). Note that, similarly to Y and X, τ may represent the combination of any number of structures and, in particular, log⁡1/p(τ) is obtained from the summation of all nested and nesting graphs.

K is a standardized metric, that in essence penalizes the amount of information obtained (calculated in the numerator) by the total information needed to describe the system (calculated in the denominator). In its basic formulation given above, *K* is a quantity between 0 and 1, which grows larger for knowledge claims that, all else equal:

(a) Achieve higher signal-to-noise ratios—smaller H(Y|X,τ).
(b) Are about broader and more accurately measured phenomena—larger H(Y).
(c) Are more general—larger $n_{y}$.
(d) Require less side-information and conditions—smaller H(X) and $n_{x}$.
(e) Are accounted for by simpler structures (systems, models, theories, etc.) and, to be observed, require simpler procedures—smaller log⁡1/p(τ).

Put simply, a study or field has a higher K value to the extent that it yields more information and requires less of it. The average K of studies and fields is expected to decrease moving from mathematics to the humanities, passing through the physical, the biological and the social sciences, because the average complexity and variability of the phenomena and methodologies increases across these domains [29,30]. However, the variability is much greater within domains than across them, and the proper unit of analysis is a ‘claim’, intended as a specific finding or findings relevant to a defined research question. Moreover, for most types of research the system underlying a knowledge claim cannot be described entirely (since most details about phenomena and methods are unknown or tacit), and therefore K values are most meaningful when measured in relative terms, between systems that are similar but differ in measurable details.

Re-arrangements of the *K* function answer specific metascientific questions, and the theory’s general prediction is that studies or fields with larger *K* values will manifest to a higher degree the properties associated with science—for example, high-*K* fields should accumulate more consistent evidence, reach higher consensus and make faster progress [13]. Since researchers will form experiences and intuitions about types of research, this prediction applies to the actual empirical properties of a system as well as to how that system is perceived to perform. For example, a published result would be expected to be more reproducible if it reported (or appeared to report) a large effect, that was obtained on numerous observations, following simple procedures, and under relatively non-restrictive conditions.

### 1.2. Predicting reproducibility with ICT

This study tests a general and a specific prediction made by ICT about reproducibility:

(1) A first, general prediction is that reproducibility forecasts will be reflected by their K value. Specifically, studies with higher K values should be perceived as more reproducible by researchers. Since K=I/D, this suggests that reproducibility predictions should be higher for studies reporting larger effect sizes (reflected in I) and lower for studies reporting more complex protocols (reflected in D), but note that this prediction is about K, the ratio between the two.
(2) ICT also makes specific predictions concerning the reproducibility of effects [13]. In particular, it posits that the I of the replication study will be an exponentially declining function of the I of the original study, due to the presence of divergences between the original and replication systems (see [13] for more details). If we assume that the complexity of the replication protocol reflects the likely number and impact of divergences, we expect reproducibility forecasts to correlate negatively with the complexity of the replication protocol alone.

## 2. Methods

### 2.1. Data from the BRI

The BRI started in early 2018 as an effort to evaluate the reproducibility of Brazilian biomedical science, following the blueprint of previous multi-centre efforts. Studies to be replicated were chosen as a representative sample of three methods that are commonly used in Brazilian biomedical science. At the time this manuscript was prepared, experiments had been completed but final results were still being compiled [14].

#### 2.1.1. Replication protocols

Experiments with the three selected techniques (MTT assay, RT-PCR and elevated plus maze) were selected from a random sample of life sciences articles between 1998 and 2017 with most of their authors based in Brazil (for more details on the selection, see https://osf.io/57f8s and https://osf.io/u5zdq). From each selected experiment, detailed information was extracted regarding the biological model, procedures, treatments and outcome measured in the target experiment. For each method, an exhaustive list of steps was used as a reference in order to have all protocols follow a standard description format.

All protocols were structured into the following homologous sections:

— Abstract: succinctly describing the essence of the experiment.
— Subjects and conditions: describing the experimental model and related details (i.e. housing for animal experiments, culture conditions for cell line experiments).
— Experimental procedures: describing the procedures that treatment and control animals/cells (as well as any additional groups) had undergone.
— Measurement procedures: describing the procedure to measure the outcomes (MTT assay, mRNA quantification or elevated plus maze (EPM) measurements).

For each of these sections, all information deemed relevant to the experiment that was available in the original article was included in the protocol. Conversely, information that was deemed potentially relevant but was missing from the original article was included as a series of questions associated with each portion of the protocol. Each of three labs performing replications was asked to answer these questions in order to fill the gaps in methodological descriptions (further details can be found at https://osf.io/gsvy2). Effect sizes of the original experiments were extracted from figures in the original articles, using the plot digitizing software GSYS (https://www.jcprg.org/gsys/ver1/gsys-e.pdf).

#### 2.1.2. Survey data

Survey participants were recruited through institutional emails and open invitations in social media, targeting mostly researchers from the life sciences. Inclusion criteria were: being above 18 years old and having previous or current experience in experimental research. Participants who met these criteria were directed to the survey, implemented via SurveyMonkey. Participants were free to select the method (MTT, RT-PCR or EPM) for which they would like to offer experimental predictions (i.e. for 20 out of 60 total experiments).

Each survey participant was shown an abstract describing the experiment (not the full replication protocol), the figure or table containing the original result, and its reported effect size and statistics. Different survey versions were created, where half of the experiments were selected at random to contain a link to the original article along with its title, publication venue, authors and affiliations, while the other half did not contain any study information. Participants were randomly assigned to different survey versions, so that different participants had access to different sets of articles.

A total of 69 participants completed the survey, including 20 from collaborating labs in the BRI. Participants from the BRI consortium did not forecast results for experiments that they were involved in replicating. For each experiment, they were asked to answer the following questions, preceded by the name used to indicate each variable:

(1) Replication probability: In your opinion, what is the probability that the replication will obtain an effect significantly different from 0 (*p* < 0.05) in the same direction as the original effect in a fixed-effects meta-analysis of the three independent replications? (0–100%)
(2) Relative effect size: How large would you expect the effect size to be in the replication, relative to the original effect size? (in per cent of the original unstandardized effect size; 0 indicates no effect, while negative numbers indicate effects in the opposite direction)
(3) Replication difficulty: How logistically and technically challenging to replicate does this experiment seem to be (relative to other laboratory experiments using EPM/MTT/RT-PCR)?
(4) Read the paper: If you were given the link to the original paper, did you open the paper and used this information for your predictions?

Participants were also asked general questions about their own expertise in the beginning of the survey, of which we report those relevant to this study:

(1) Knowledge (theoretical): How would you rate your theoretical knowledge of the (EPM/MTT/RT-PCR) technique?
(2) Knowledge (practical): How much practical experience do you have with the (EPM/MTT/RT-PCR) technique?
(3) Knowledge (statistical): How would you rate your knowledge of basic statistics and research methodology?

Replication protocols and survey data from the BRI are currently under embargo and will be made available once replication results are published.

### 2.2. Calculating *K* values

All the variables necessary to quantify the *K* function in equation (1.1) were obtained as described in [24]. Briefly summarized, the terms of equation (1.1) were defined and measured as follows, in all cases expressing a quantity of bits:

— **Input:** H(X)=1.
  *Explanation:* Since all studies represent experiments with random allocation to a treatment and a control group, the input is simply the information generated by allocating each unit to one of the two groups with equal probability. This corresponds to the Shannon entropy of a binary random variable with uniform distribution, which is 1 bit.
— **Explanandum:** H(Y)=1
  *Explanation:* Since the input generates one bit of information, the maximum amount of information that can be extracted from the explanandum is also one bit. This is the information required to fully separate treatments from controls. If the input (allocation to treatment versus control) works perfectly well, it will unambiguously segregate the units in these two groups along the values measured in the explanandum. This would correspond, for example, to having all the outcome values of the treated units falling above those of the controls.
— **Repetitions:** $n_{Y}=n_{X}=n$
  where n is the sample size.
  *Explanation:* As explained in the introduction, the repetitions $n_{y},n_{x}$ are intended to express the extent to which the knowledge can be applied. Theoretically, this is the number of every possible individual or object to which this same experiment can be applied. However, applying the concept in a narrow sense, we shall consider the sample size to be a proxy of this quantity.
— **Residual ignorance:** $H(Y|X,\tau )=\frac{1}{n}\times \left[n_{C}H(C)+n_{T}H(T)\right]$
  where $H(C)=-p(Y_{C}<m_{Y})\;\log\;p(Y_{C}<m_{Y})-p(Y_{C}\geq m_{Y})\;\log\;p(Y_{C}\geq m_{Y})$ and $H(T)=-p(Y_{T}<m_{Y})\;\log\;p(Y_{T}<m_{Y})-p(Y_{T}\geq m_{Y})\;\log\;p(Y_{T}\geq m_{Y})$ are, respectively, the conditional entropies of the outcome for treatment and controls, calculated from the probabilities to fall above or below the overall median $m_{Y}$ of the outcome.
  *Explanation:* We showed above that the explanandum is H(Y)=1 bit of information, and maximum information is attained when treatment and controls are completely segregated. The residual ignorance evaluates the extent to which such a segregation is attained. We take the overall median (which splits the sample in half) and calculate the binary entropy values for the outcome of treatment and controls separately, and average them. If the effect is null, then both treatment and control units have equal probability to fall above and below the median $m_{Y}$, yielding H(Y|X,τ)=1 and therefore I=0 in equation (1.1). Conversely, if the effect is maximal, then all treatments are on one side of the median, and all controls on the other, so the entropy for each group is 0, which gives H(Y|X,τ)=0, yielding the maximum value of I attainable by that system.
  Ideally, the entropies could be derived directly from the frequency distribution of observations. However, since the raw data for the primary studies were not available, H(C),H(T) were calculated from the summary data (mean, s.d. and sample size), assuming that both treatment and control values of Y were normally distributed and using the weighted mean of the two groups in place of the median, i.e. $m_{Y}=\left(n_{T}m_{t}+n_{C}m_{C})/(n_{T}+n_{C}\right)$.

— **Theory/methodology:** log⁡1/p(τ)=D(τ) where τ is a nested hypergraph that describes the replication protocol, and D is a recursive function that calculates the description length of each level of each branch of the hypergraph. The D function is defined as follows:

(2.1) $$\begin{array}{r} D(\tau )=0\;\text{if }\tau \in \emptyset \\ D(\tau )=\log\frac{1}{p(\tau )}+\sum_{\tau_{i}\in V,E}D(\tau_{i})\;\text{otherwise,} \end{array}$$

in which V,E are sets containing the descriptions of the vertices and edges of the graph—these descriptions are simply other graphs. The description length of each graph is calculated as

(2.2) log⁡1p(τ)=log⁡(dar )+log⁡(dmv1,mv2...mvp)+log⁡(rme1,me2...meq)−∑i=1qlog⁡s(ei),

in which d is the number of vertices, r the number of edges (relations between vertices), a is the maximum arity of the relations, $m_{v1}...$ are the multiplicities of the labels v1... of the vertices, $m_{e1}...$ those of the edges (relations) and s() gives the number of bits saved for each relation $e_{i}$ that is symmetric (in which two or more vertices inputting into that relation can be swapped).
*Explanation:* The τ can be described as a recursive ‘structure of structures’, a ‘relation of relations’ with unlimited recursive depth. Mathematically, such an object can be represented as a nested labelled hypergraph, that is, a graph whose edges are subsets of the graph’s nodes, and in which both nodes and hyper-edges carry ‘labels’ that are pointers to lower-level (nested) descriptions. These lower-level descriptions are in turn other hypergraphs, and so on.
Equation (2.1) quantifies the description length of each composing hypergraph, as a Shannon code log⁡1/p(), starting from the top-most level, conventionally indicated as τ, and moving its way down each nested element $\tau_{i}$ of each level in the graph, until no further lower-level description is given. For each graph with d vertices, r relations (hyperedges) of maximum arity a, with labels of vertices and relations distributed as a multinomial distribution with multiplicities m and with some edges (relations) characterized by symmetries s(e), the optimal encoding is calculated as the logarithm of the size of the set of all possible equivalent graphs, which is given by the combinatorial calculation in equation (2.2). Thus, the total description length of the theory/methodology is obtained as the sum of the encodings $\log\;1/p(\tau_{i})$ of all graphs $\tau_{i}$ that comprise the description.

### 2.3. Quantifying protocol complexity

In order to calculate the D value of a study’s methodology, it is necessary to translate that methodology into a hypergraph. This requires defining a suitable scheme that allows us to compare the complexity of studies by identifying a common high-level structure and then measuring the variable length of the lower levels.

To quantify the *D* of each BRI replication, we parsed the text of its replication protocol according to a general, higher-level scheme similar to that proposed in [31] for randomized experiments. In the graphs that follow, rectangles represent vertices, romboids are edges representing relations of arity equal to the number of vertices connecting to them, arrows represent the edge direction (when present) and the boxing of a graph within a rectangle represents the nesting relation. Note that the labels of vertices and edges have no intrinsic meaning and are merely pointers to lower-level descriptions.

Starting from the highest level, the τ is described as follows:


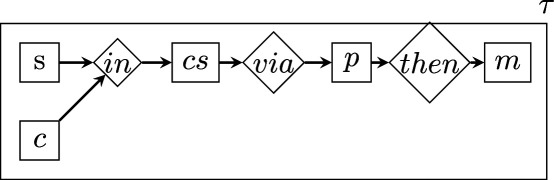


where


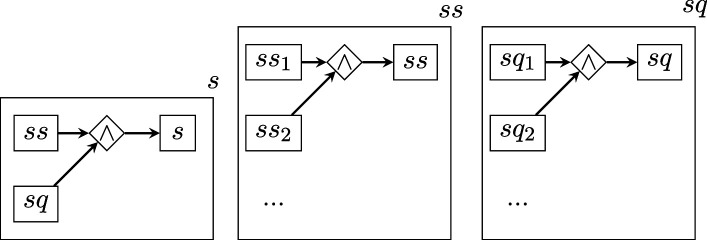


describes the subject conditions ss (the set of properties required of animals, cell lines, etc.) and the questions sq that BRI asked each laboratory to answer about subjects, in order to fill in potentially relevant details;


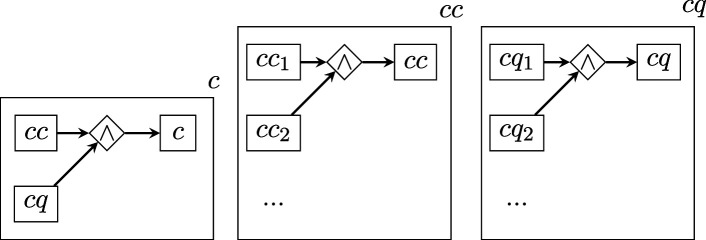


describes the experimental conditions in terms of the list of conditions themselves cc and the questions cq about them;


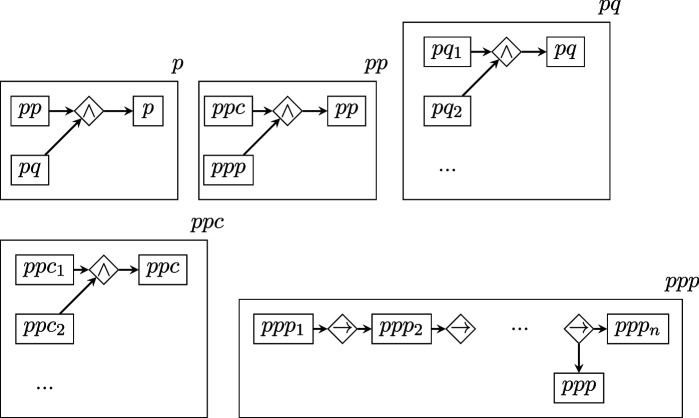


describes the experimental procedure component p, as a combination of procedure details pp and questions about missing details pq, with procedure details consisting in procedure conditions ppc and precedural steps ppp; and


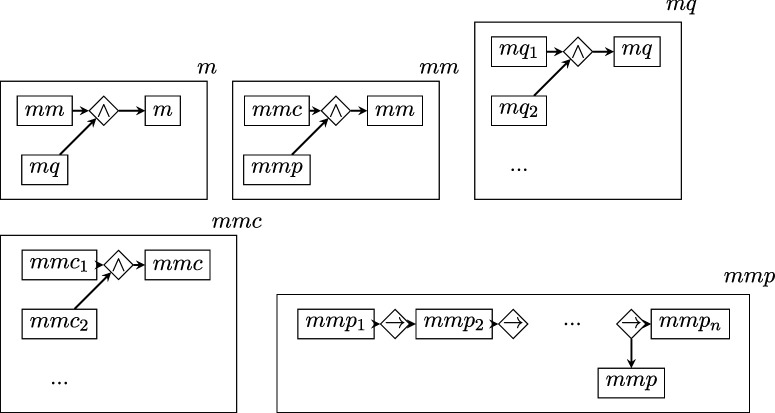


describes the measurement component m with the same scheme as p.

The total description length of this structure is obtained by applying the recursive function 2, which sums up the description lengths $\log\;1/p(\tau_{i})$ of each level within each branch of the graph.

Note that all the graph levels above the bottom one are the same for all studies. Therefore, their D value is a constant that will not affect estimations of relative complexity and can be ignored.

The bottom levels ss, sq, cc, cq, ppc, ppp, pq, mmc, mmp, mq are instead structures of varying shape and size, which yield a D value that varies across protocols, where higher values indicate greater protocol complexity (e.g. more experimental conditions specified, or procedures requiring more steps).

As shown above, these lower-level structures have two possible shapes: (i) symmetric ‘stars’, connecting all concepts via a single logical AND (∧) relation; (ii) non-symmetric ‘chains’ of steps connected by THEN (→) relations. Each element in these stars or chains (e.g. $s_{1}$, $s_{2}$...$ccc_{1}$, $ccc_{2}$ etc.) contains a ‘concept’, that is a chunk of separate information, derived directly from parsing the text of the protocol (this procedure is described further below).

The description of the lowest levels of the graph technically consists in portions of the text itself. In our main analyses, these lowest-level elements were given a standard description length of 1 bit, thereby ignoring any additional information that might be contained in the length of the text.

Electronic supplementary material, figure S1 shows an example graph describing a protocol.

### 2.4. Parsing the replication protocols

#### 2.4.1. Hand-parsing method

The text of each protocol was first subdivided in the bottom-level categories described above (e.g. subject conditions, experimental conditions, questions, etc.), and the strategy for parsing the protocol texts was elaborated by DF based on theoretical principles and heuristics. This strategy required parsing choices that were specific to the type of details and reporting offered in the BRI replication protocols. For example, sentences describing the chemical components of an off-the-shelf PCR mix were not parsed, but those for a generic cell culture medium that the experimenter might need to prepare were parsed. This is because the latter chunks encode steps that the replicator actually has to follow.

This logic was translated into a set of instructions that were used by KN and PT to measure inter-coder reliability. These instructions are reported below *verbatim* (except for a few grammatical and typographical corrections):

— The scope is to parse the instructions into self-contained (i.e. mutually non-redundant) items of information that the experimenter needs to follow. This entails breaking the text into natural sentences, but then breaking it down (or sometimes recombining it) further, into separable concepts/steps, following the conventions below.
— ‘Self-contained item of information’ means non-redundant (e.g. ‘we incubated the plates in anaerobic conditions’ describes a single concept—that cells need to be kept in a certain condition—so it all goes together).
— If two items are non-redundant, then they are separable (e.g.‘mice in pairs of different sexes’ describes two conjoint, separable, concepts—‘mice in pairs’ and ‘of different sexes’).
— The number of items is a concept.
— Ranges, error terms, and redundant info (e.g. molar concentration and percentage in mixture) go together.
— Temperature + error are a concept.
— Reagent name and its quantity (e.g.‘5% CO2’) is a concept.
— Time of day is a concept.
— Time occurring between one step and the next step is a concept, i.e. a step (e.g. ‘after five hours we sacrificed the mouse’).
— The sizes of an item are a concept, aggregating all dimensions (e.g. ‘40 cm high walls’, ‘5 × 5 platform’).
— Chemical mixtures are separated by component, except for stock (pre-prepared) PCR/RNA mixtures, see below. The idea is that we separate mixtures that have to be prepared, and so each component is a step in preparing the experiment.
— In conditions (s, ppc, mmc): break each individual item of information (e.g.‘male’, ‘Swiss’, ‘mice’, ‘25 + 5 g of weight’).
— In processes, the header of a step is a separate step/concept if it contains time info (e.g. ‘Experimental day 10: forced feeding treatment’).
— In processes: description of the control is not separated from description of treatment (since they count for 50% the total steps actually performed, e.g. ‘Injected with 10% NHCL, whereas controls were injected with saline’).
— In PCR/RNA procedures, we only break down [the text] in global steps, each step entailing something that the experimenter actually has to do (e.g. ‘shake for 20 s’ ‘place on ice’...). Whereas anything that is pre-defined and/or automatic (e.g. the PCR mix provided by the company, or the sequence of temperatures and times in the PCR process) counts as a single step. Similarly, specifications about the machine used or other stock products (e.g. list of primers) are a single chunk, separate but not further broken down.

All protocols and abstracts were parsed by DF, and the parsing was blind to any prediction and replication results.

To assess the reliability of the scheme, KN and PT coded nine protocols (three for each method type) after a brief explanation and training, with the list of instructions given above. The D(τ) of the resulting graphs had a correlation of *r* = 0.79, and discrepancies were mainly found within one protocol due to explainable (and in principle resolvable) differences in the logic of text parsing. This suggests that the method is replicable in principle and capable of yielding sufficiently consistent measurements across raters, with adequate training.

### 2.5. Calculating the K value

The *K* value of each study was calculated by plugging in the measurements made above in the formula:


(2.3) $$K=\frac{1-\frac{1}{n}[n_{C}H(C)+n_{T}H(T)]}{1+1+\frac{\log1/p(\tau )}{n}},$$

where $n=n_{C}+n_{T}$ and


log⁡1p(τ)=76.2+D(ss)+D(sq)+D(cc)+D(cq)+(2.4)D(ppc)+D(ppp)+D(pq)+D(mmc)+D(mmp)+D(mq),

in which the first number encodes the scheme itself and, being constant across all studies, does not affect results, and the remaining quantities are the description lengths of lower-level graphs, which vary across studies.

### 2.6. Analyses

For each included study, we calculated the average predicted probability of replication (measured as a percentage, with two decimals) and the predicted relative effect size (expressed on a scale from 0 to ∞). Both these variables were treated as continuous (interval scale) variables.

To test prediction 1, we ‘log-odds transformed’ K as:


(2.5) $$\begin{array}{lcr} & \log\;\frac{K}{1-K} & \end{array}$$

in order to extend the domain from (0,1) to (−∞,+∞). In practice, this makes a small difference relative to regressing on K, since K values were small. Results with the untransformed K are also provided in §3 (table 1).

**Table 1.** Multiple regression results comparing the explanatory power of different variables and their transformations and combinations on ratings of replication probability (left) and relative effect size (right). From left to right, columns a-h show different regression analyses using different variables to predict each of the two outcomes. MTT and PCR are dummy variables representing study type with EPM as the reference category; SMD refers to the standardized mean difference for the experiment; difficulty refers to the subjectively rated technical difficulty of conducting the replication; n is the study sample size; ls, dn,and r are functions that, with calculations shown in the table, progressively approximate the structure of K and KLO. One, two and three asterisks indicate P < 0.05, P < 0.01, P < 0.001.

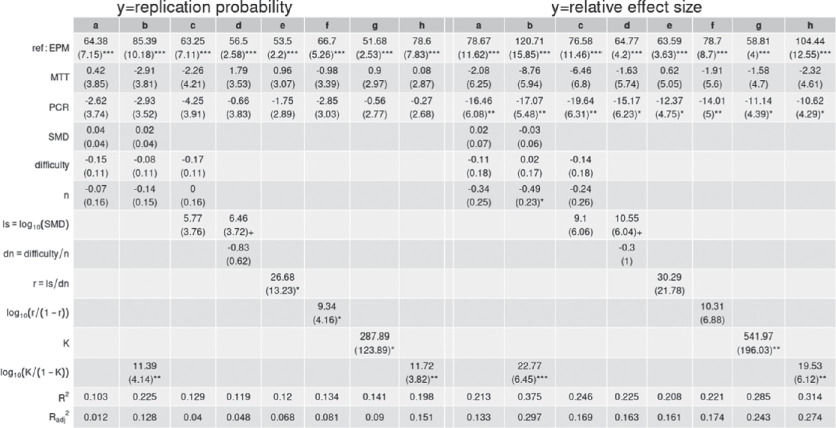

All statistical estimates were obtained with a standard ordinary least squares regression, with models specified in the text. Unless otherwise specified, all regression estimates reported in the text are partial associations controlling for study method. All main analyses and robustness/sensitivity analyses are reported in the R script included as electronic supplementary material. The R script also reports, as commented text, the results of all secondary or robustness tests not directly reported in §3.

## 3. Results

**Figure 1.**
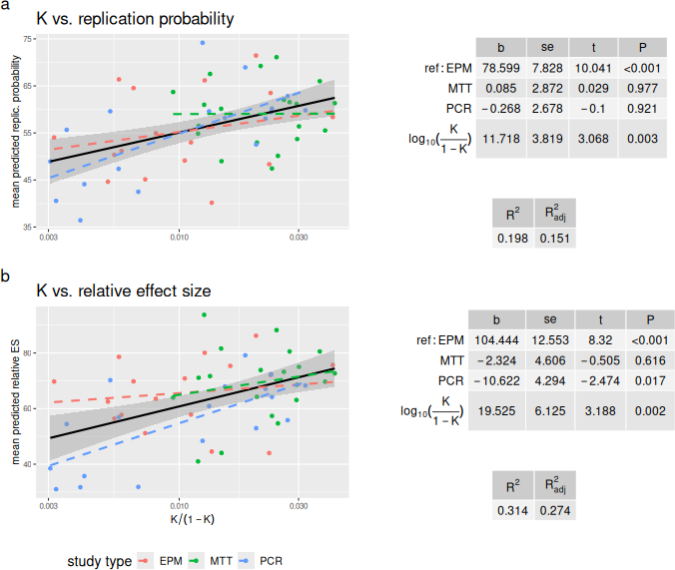
Relation between *K* values and subjective ratings of replication probability (a) and relative effect size (b). *K* is ‘log-odds-transformed’, yielding the measure called ‘KLO’ in the text. Scatterplots show univariate relations, for each study method (coloured dashed lines) and overall (black). Grey area shows the 95% ~confidence interval for the overall regression line. Tables give results of ordinary least squares multiple regression (showing slope, standard error, *t*-value and *p*‐value) controlling for study method (EPM = elevated plus maze, used as the reference category, MTT = MTT assay, PCR = reverse-transcriptase PCR). See text for further details.

We found that *K* was a substantive predictor of reproducibility ratings. In univariate analyses, the log-odds-transformed *K* (henceforth, KLO) explained 20% or more of the variability of ratings (ratings about replication probability: b=11.88±3.29,p<0.001, $R^{2}=0.20$, $R_{adj}^{2}=0.18$; ratings about relative effect size: b=21.95±5.61,p<0.001, $R^{2}=0.22$, $R_{adj}^{2}=0.21$). After controlling for the three experimental methods (which is an important confounder, since methods vary in average description length and had different raters), KLO retained a similar, highly significant effect, with one unit increase in KLO associated with around 0.12 additional predicted probability of replication and 20% larger predicted relative effect size (figure 1, results with non-transformed *K* values are shown in table 1). A model that only included method as a fixed effect explained little of the variance in replication probability ratings ($R^{2}=0.05$, $R_{adj}^{2}=0.01$) and around 18% of the variance in relative effect size ratings ($R^{2}=0.18$, $R_{adj}^{2}=0.15$), suggesting that KLO increased the variance explained by between 12% and 15%.

**Figure 2.**
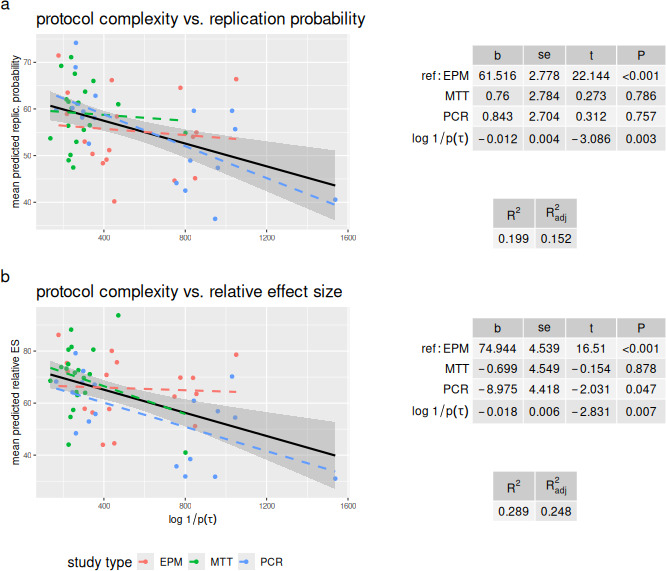
Relation between the descriptive complexity of protocol (calculated by applying equation (2.1) to the graphical description of each protocol) and subjective ratings of replication probability (a) and relative effect size (b). Scatterplots show univariate relations, for each study method (coloured dashed lines) and overall (black). Grey area shows the 95% ~confidence interval for the overall regression line. Tables give results of ordinary least squares multiple regression (showing slope, s.e., *t*-value and *p*‐value) controlling for study method (EPM = elevated plus maze, used as the reference category, MTT = MTT assay, PCR = reverse-transcriptase PCR). See text for further details.

Protocol complexity alone was also negatively associated with reproducibility ratings (figure 2). In univariate analyses, protocol complexity explained 18% or more of the variability (ratings about replication probability: b=−0.01±0.003,p<0.001, $R^{2}=0.20$, $R_{adj}^{2}=0.18$; ratings about relative effect size: b=−0.02±0.006,p<0.001, $R^{2}=0.22$, $R_{adj}^{2}=0.21$). Inspection of the data shows a potentially influential point (i.e. one of the PCR experiments, with very large complexity, figure 2), but removing this point did not alter the results substantially (ratings about replication probability: b=−0.01±0.004,p=0.005, $R^{2}=0.14$, $R_{adj}^{2}=0.13$; ratings about relative effect size: b=−0.02±0.006,p=0.003, $R^{2}=0.15$, $R_{adj}^{2}=0.14$).

**Figure 3.**
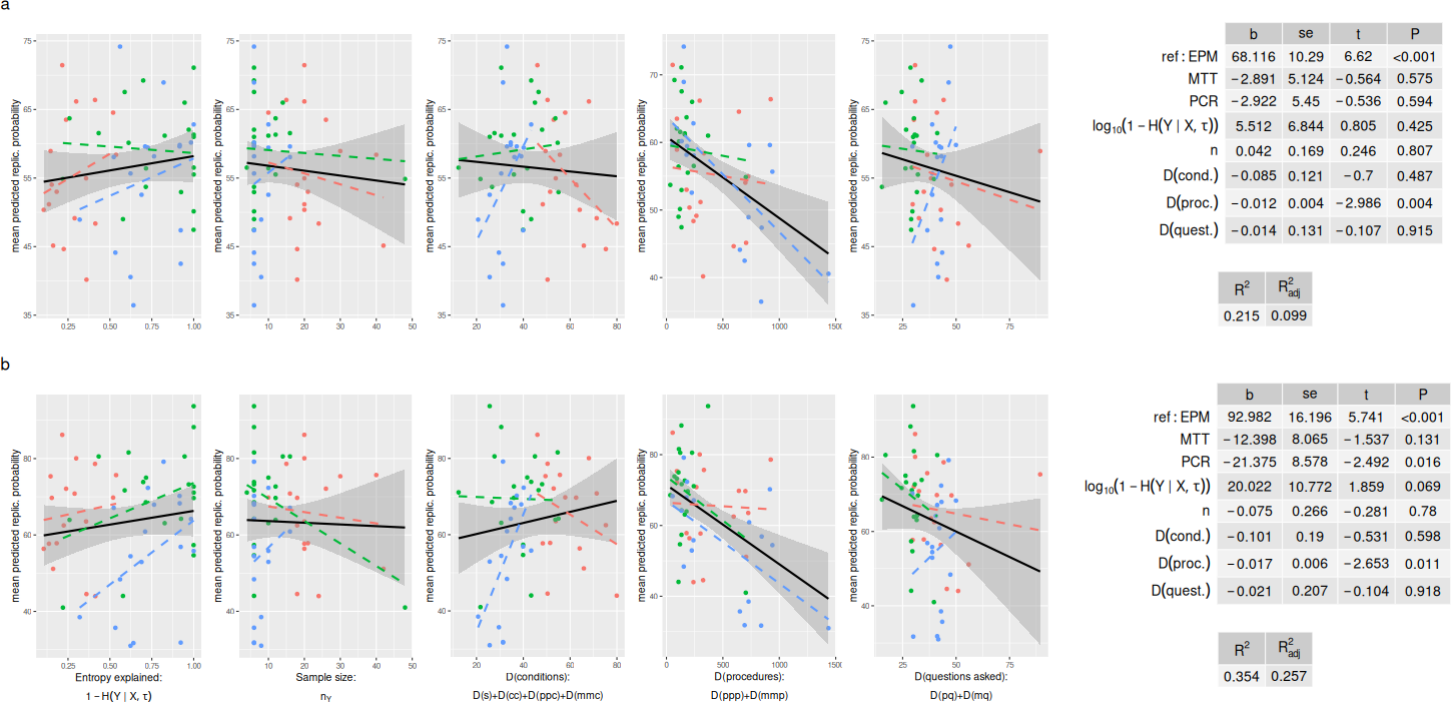
Scatterplots and multiple regression results assessing the relation between various components underlying the *K* function and subjective ratings of (a) replication probability or (b) relative effect size. The components, from left to right, are: entropy explained (the numerator of K), the sample size of the original study and the complexity (description length) of experimental conditions, experimental procedures and methodological questions asked, respectively—with the latter reflecting details that were missing in the original publication and were deemed important to specify in the replication protocol. Scatterplots show univariate relations, for each study method (coloured dashed lines) and overall (black line), on a logarithmic scale. Grey area shows the 95% ~confidence interval for the overall regression line. Tables on the right report the corresponding multiple regression estimates.

We explored the nature of these associations by regressing replication ratings against separate components of K and τ. Replication ratings were positively but not significantly correlated with the entropy explained and were negatively correlated with the complexity of components of the experimental and measurement procedures (figure 3). A full breakdown of the methodology suggests that the strongest correlation is with the length of measurement procedures (mmp), whereas there is a weaker and positive association with the number of measurement conditions (mmc, see electronic supplementary material). This suggests that multiple components of the K function might contribute to explaining the variance in ratings.

We also calculated KLO values from the protocol summaries that were shown to participants and were the source of ratings, rather than those of the full protocol. These KLO values were positively but not significantly associated with ratings about replication probability and were highly significantly associated with ratings about relative effect size (electronic supplementary material, figure S2).

We tested whether ordinary metrics of effect size (i.e. standardized mean difference, SMD) and ratings of replication complexity (i.e. the participant’s own rating of the difficulty of the experiment) would have similar or stronger effects. SMD was highly correlated with the entropy explained (Spearman’s rank-correlation: ρ=0.93, p<0.001) and perceived difficulty was significantly associated with protocol complexity log⁡1/p(τ) (ρ=0.65, p<0.001) and particularly with the complexity of procedures (controlling for methodology and all components of τ, b=0.2.37±0.00, p<0.001, see electronic supplementary material for further details). However, the SMD and difficulty ratings were weakly associated with replication ratings and, even controlling for them, KLO remained a significant predictor (table 1, columns a, b). Transformations and integrations of SMD and difficulty into a structure analogous to KLO made them progressively better predictors of ratings (table 1, columns c–f). However, *K* was still generally the strongest predictor, particularly when transformed as KLO (table 1, columns g, h).

The level of expertise and knowledge possessed by raters may be a significant moderator of these effects. When KLO was regressed against the scores of participants who self-rated their knowledge of the method used in the replication as ‘good’ or ‘excellent’, the partial association was as large or larger than that observed for the whole sample (when restricted to participants with high self-rated ‘practical knowledge’: b=16.36±4.22, p<0.001, $R^{2}=0.31$, $R_{adj}^{2}=0.27$ for ratings about replication probability and b=20.77±6.20, p<0.001, $R^{2}=0.28$, adjusted $R^{2}=0.24$ for ratings about relative effect size. High correlations were also obtained when restricting the sample to respondents with high ‘theoretical knowledge’ and ‘statistical knowledge’, see electronic supplementary material). Conversely, ratings of participants who reported their knowledge of the methods to be below ‘good’ were less strongly associated with KLO (for ‘practical knowledge’, ratings about replication probability: b=2.90±4.84, p=0.55, $R^{2}=0.13$, $R_{adj}^{2}=0.07$; ratings about relative effect size: b=15.13±9.32, p=0.111, $R^{2}=0.26$, $R_{adj}^{2}=0.22$), although this difference was reduced if one of the methods, MTT, was excluded from the analysis (see electronic supplementary material).

If we restricted the analysis to participants who reported having examined the full text of the article in which the original claim was made, the predictive power of *K* was weaker (replication probability ratings: b=3.52±6.03, p=0.562, $R^{2}=0.02$, $R_{adj}^{2}=-0.03$; relative effect size ratings: b=15.44±9.38, p<0.106, $R^{2}=0.15$, $R_{adj}^{2}=0.10$). No such reduction was observed among authors who had not read the paper (respectively, b=12.53±4.09, p=0.003, $R^{2}=0.21$, $R_{adj}^{2}=0.16$; b=19.38±6.62, p=0.005, $R^{2}=0.29$, $R_{adj}^{2}=0.25$).

## 4. Discussion

Metascientific studies had repeatedly shown that experts can predict the reproducibility of studies above chance, but how these experts form these judgements is largely unknown. This study shows that a theoretical framework for metascience based on information compression may contribute to the answer. In particular, our results suggest that expert judgements might be partially determined by the number of bits of information yielded by a study and the number of bits needed to describe it. Whether alone or combined in a ratio, which constitutes the variable K, these factors were more strongly correlated with researchers’ judgements than ordinary, non-entropy-based metrics—respectively, the SMD and the expert’s own rating of how difficult the experiment is (table 1).

**Figure 4.**
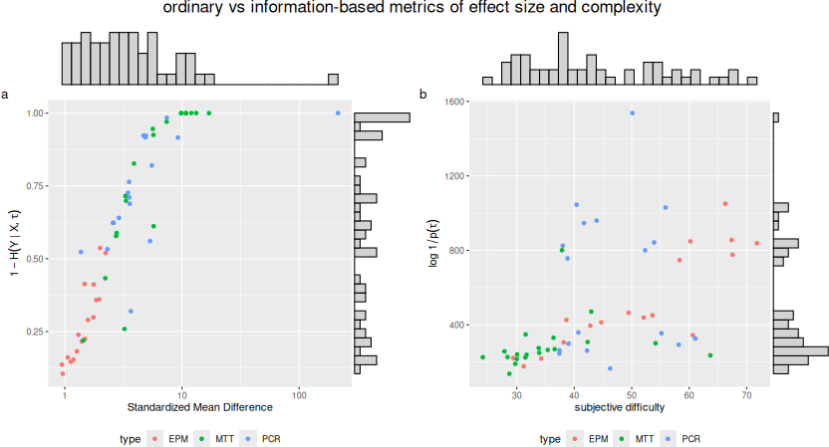
Scatterplots and histograms comparing quantities used to calculate *K* to more conventional equivalents: (a) effect size measured as SMD, on a log scale, versus the numerator of *K*, which measures effect size in terms of bits of Shannon entropy; (b) subjective ratings of methodological difficulty versus the key component at the denominator of *K*, which measures the complexity of the methodology in terms of a ‘description length’ function *D* applied to a conceptual graph. See text for more details.

Why would *K* outperform analogous, more conventional measures? We speculate that it may be due to a combination of structure and metrics properties. The structure of K was derived from an intuitive logic: if studies yield finite amounts of information and vary in complexity, in order to make accurate between-study comparisons, we must standardize their information yield, and so we divide it by the information needed to describe their underlying system [13]. It is plausible that the same intuitive logic is followed by human raters when evaluating the strength of a finding. In support of this hypothesis, we found that a metric constructed by dividing SMD by difficulty ratings and log-odds transforming, thereby creating a structure analogous to *K*, produced a better predictor of ratings than SMD and difficulty alone (table 1c–f). However, *K* was still the strongest predictor (table 1g,h), suggesting that, in addition to K’s particular structure, the use of information quantities may also play a role. In particular, the entropy-explained metric at the numerator of *K* ‘compresses’ the values of SMD: it is exponentially more sensitive to small values of SMD, and much less skewed than SMD (figure 4a). It is plausible that human raters evaluate effect sizes in a similar way, paying close attention to how SMDs change when they are small, and considering large values of SMD as essentially equivalent. The metric at the denominator is positively associated with subjective ratings of difficulty (figure 4b). *D* is calculated directly from the protocol, and it grows with the number and diversity of steps involved in an experiment. Therefore, it is plausible that *D* constitutes a more fine-grained measure of the difficulty of correctly replicating a result, which would explain its independent and significant correlation with reproducibility predictions (figure 2).

Therefore, our results suggest that *K*, a principled metric that embodies a philosophical assumption about the nature of scientific knowledge, may reflect part of the cognitive processing that scientists use, consciously or not, when forecasting the reproducibility of a study. Like *K*, scientists might implicitly assess the amount of information explained by a result relative to the information (complexity) of methods that underlie it.

Among the various components of replication protocols, we found that expert predictions were mainly correlated with the complexity of measurement procedures. We hypothesized that the lack of association with some of the components of the methodology could be due to the fact that participants had not read the full replication protocol but merely a summary. Surprisingly, however, *K* values based on the summary alone were less strongly associated with predictions than *K* values based on the entire protocol, especially for replication probability ratings (electronic supplementary material, figure S2). This suggests that the protocol summaries were used by participants as proxies of the actual complexity of the study. In support of this hypothesis, we found that predictions by scientists who self-rated as more knowledgeable of the methods were more strongly associated with K, suggesting that expertise allowed scientists to better estimate, from the protocol summary, how complex the actual experiment was going to be.

Secondary analyses suggest that predictions might be less strongly correlated with *K* among raters who had read the original publication, as would be expected if the full text of the original article contained additional information that the researchers have used to make predictions. This information might include further scientific details about the methods and effects involved, including other results reported in the study, and it might also include indirect cues of scientific quality, such as the identity of the authors, their institutional affiliation, and the journal in which the study was published.

It should be stressed that, due to the highly novel nature of this approach, the significance of these results is mainly theoretical. Work in progress will assess the ability of ICT to forecast actual reproducibility, whether it outperforms human raters at this task, and whether similar results are obtained in other types of research.

The methods presented in this study should be considered a proof of concept, to be built upon and improved. They show that it is useful and possible to quantify the complexity of study descriptions, but the best approach to doing so remains to be established. Whilst not technically infinite, the number of possible schemes (i.e. different graphical structures, categories, types of relations, etc.) that may be considered in order to parse a text is large, and different parsing logics may be able to capture different features of the complexity of a study, while missing others. More sophisticated methods of quantifying graph complexity might also be considered, and it remains to be established to what extent a single approach to measuring complexity yields useful results across different types of research. It is likely that different schemes and methods might be required to capture the relevant features of different systems. Another important limitation of our methods is the hand-parsing of text, which introduces noise and arbitrariness. Ideally, future research should develop approaches that are mostly or entirely automated, via the use of Natural Language Processing techniques and possibly involving the analysis of texts by large language models.

In conclusion, while the practical significance of our findings remains to be established, we emphasize their theoretical and philosophical relevance. Our finding that ICT’s key metric reflects how scientists rate the potential reproducibility of experiments helps to explain how expert forecasts about reproducibility are formed, and it supports the hypothesis that scientific knowledge can be usefully understood as a process that strives to maximize information compression and consilience [13,15,21,22,32,33].

## Data Availability

Raw data is included as electronic supplementary material [34].
